# Transcriptome Data Reveal Syndermatan Relationships and Suggest the Evolution of Endoparasitism in Acanthocephala via an Epizoic Stage

**DOI:** 10.1371/journal.pone.0088618

**Published:** 2014-02-10

**Authors:** Alexandra R. Wey-Fabrizius, Holger Herlyn, Benjamin Rieger, David Rosenkranz, Alexander Witek, David B. Mark Welch, Ingo Ebersberger, Thomas Hankeln

**Affiliations:** 1 Institute of Molecular Genetics, Johannes Gutenberg-University Mainz, Mainz, Germany; 2 Institute of Anthropology, Johannes Gutenberg-University Mainz, Mainz, Germany; 3 Josephine Bay Paul Center for Comparative Molecular Biology and Evolution, Marine Biological Laboratory, Woods Hole, Massachusetts, United States of America; 4 Institute for Cell Biology and Neuroscience, Goethe-University Frankfurt am Main, Frankfurt, Germany; University of Muenster, Germany

## Abstract

The taxon Syndermata comprises the biologically interesting wheel animals (“Rotifera”: Bdelloidea + Monogononta + Seisonidea) and thorny-headed worms (Acanthocephala), and is central for testing superordinate phylogenetic hypotheses (Platyzoa, Gnathifera) in the metazoan tree of life. Recent analyses of syndermatan phylogeny suggested paraphyly of Eurotatoria (free-living bdelloids and monogononts) with respect to endoparasitic acanthocephalans. Data of epizoic seisonids, however, were absent, which may have affected the branching order within the syndermatan clade. Moreover, the position of Seisonidea within Syndermata should help in understanding the evolution of acanthocephalan endoparasitism. Here, we report the first phylogenomic analysis that includes all four higher-ranked groups of Syndermata. The analyzed data sets comprise new transcriptome data for *Seison* spec. (Seisonidea), *Brachionus manjavacas* (Monogononta), *Adineta vaga* (Bdelloidea), and *Paratenuisentis ambiguus* (Acanthocephala). Maximum likelihood and Bayesian trees for a total of 19 metazoan species were reconstructed from up to 410 functionally diverse proteins. The results unanimously place Monogononta basally within Syndermata, and Bdelloidea appear as the sister group to a clade comprising epizoic Seisonidea and endoparasitic Acanthocephala. Our results support monophyly of Syndermata, Hemirotifera (Bdelloidea + Seisonidea + Acanthocephala), and Pararotatoria (Seisonidea + Acanthocephala), rejecting monophyly of traditional Rotifera and Eurotatoria. This serves as an indication that early acanthocephalans lived epizoically or as ectoparasites on arthropods, before their complex lifecycle with arthropod intermediate and vertebrate definite hosts evolved.

## Introduction

Increasing amounts of sequence data combined with elaborated phylogenomic analyses are constantly refining our view on the metazoan tree of life (e.g. [1]–[7]). However, some taxonomic groups are still not well represented in terms of taxa and data availability, leaving many open questions concerning their evolutionary relationships and character evolution. Within Protostomia, a crucial question is the monophyly and placement of the hypothetical taxon Platyzoa [8], which unites Platyhelminthes (flatworms) with other mostly microscopic, worm-shaped animals like Gnathifera, and, possibly, Gastrotricha [9]. Hence, this taxon may comprise pseudo- and acoelomate animals, which for the most part develop without metamorphosis. In some groups, however, parasitic lifestyles exist including parasitizing larval stages (Neodermata within Platyhelminthes and Acanthocephala within Gnathifera).

In recent phylogenomic studies only one or at best a few putative platyzoan subtaxa were represented, most of them by only one or two species (e.g. [1]–[3], [6], [7], [10]). The resulting trees revealed unstable and contradictory positions for individual taxa and did not produce reliable support for or against the Platyzoa hypothesis (see e.g. [2], [3], [5], [11], [12]). Possible reasons included long-branch attraction (LBA) phenomena [2] and significantly deviating amino acid compositions [5], leading to poor leaf stability indices [3] for the corresponding taxa. These shortcomings can hopefully be overcome by increasing taxon and sequence data coverage [13], [14] for putative platyzoan animals, many of which are notoriously difficult to obtain in sufficient amounts.

As a further step towards addressing these superordinate phylogenetic problems, the current study has its focus on resolving the debated internal phylogeny of the taxon Syndermata, which is particularly interesting as a model for the evolution of parasitism. Syndermata unites Monogononta, Bdelloidea and Seisonidea (traditionally subsumed as “Rotifera”) with the morphologically very distinct Acanthocephala. Other authors subsume Acanthocephala within extended Rotifera [15]. Leaving aside this semantic issue, we join Garcia-Varela and Nadler [16], Min and Park [17], Minelli [18], Gazi et al. [19], Lasek-Nesselquist [20] and refer to bdelloids, monogononts, seisonids, and acanthocephalans as Syndermata. A close relation between these taxa was originally inferred from shared morphological traits such as a syncytial epidermis with an electron dense internal layer and sperm morphology [21]–[25]. Additional support for a close phylogenetic relationship of acanthocephalans and at least some of the traditional rotiferan taxa came from analyses of single- and multigene molecular data [2], [6], [10]–[12], [16], [26]–[33] as well as from analyses of combined molecular and morphological data [34], [35].

While syndermatan monophyly has received strong support, the internal syndermatan phylogeny is still not fully resolved. Morphological characters uniting the three traditional rotiferan taxa Seisonidea, Monogononta, and Bdelloidea are scarce. This is mainly due to their differences in lifestyle, morphology, and reproduction. The four presently described species of Seisonidea live epizoically, partly even ectoparasitically, on marine crustaceans of the leptostracan genus *Nebalia* [36]–[39]. In contrast, most bdelloids and monogononts are free-living aquatic animals capable of active swimming, employing a ciliated apical structure named the corona or wheel-organ. The wheel-organ in seisonids, however, is reduced or rudimentary [40], [41]. Seisonids are additionally distinguished from monogononts and bdelloids by a specific mastax and trophi structure as well as a protrusible neck [22], [41], all of which might have emerged in the context of nourishment in their epizoic, potentially ectoparasitic lifestyle. The three “rotiferan” taxa also differ with respect to their reproduction: While seisonids are strictly bisexual with well-developed males that co-occur with females throughout the year, bdelloids and monogononts are capable of parthenogenesis and have dwarf (Monogononta) or even no males (Bdelloidea) [42]. The fourth syndermatan taxon, Acanthocephala, comprises worm-like endoparasites with a complicated life cycle including an arthropod intermediate host and a vertebrate definite host. Sexes are separate, with adult females being larger than males, and reproduction is exclusively sexual. As in other parasites, the morphology of acanthocephalans is highly modified. For instance, the mastax and intestinal tract are absent and nutrient uptake occurs via epidermis. Moreover, adult acanthocephalans possess a retractable hooked proboscis for anchoring to the intestinal wall of the host (e.g. [25], [43]).

**Figure 1:**
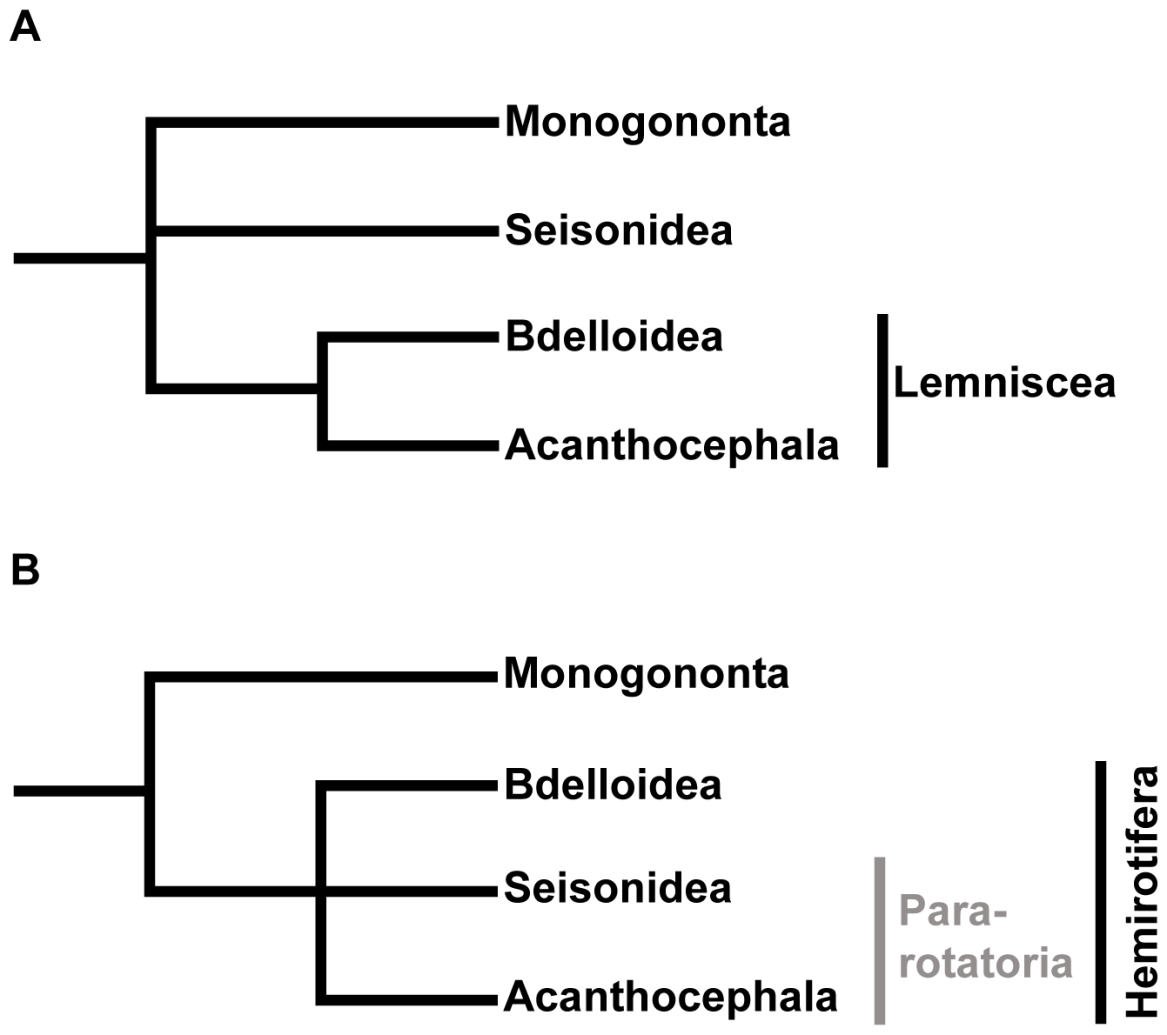
Two alternative hypotheses for the internal syndermatan phylogeny. Alternative hypotheses for the internal syndermatan relationships are (A) the morphology-based Lemniscea hypothesis [45], and (B) the Hemirotifera hypothesis [35], which is mainly based on molecular data. The latter did not specify the sister group of Acanthocephala. Support for a potential sister group relationship of Acanthocephala and Seisonidea (Pararotatoria) comes from morphological data [22], [23] and analyses of partial 18S rRNA sequences [26].

Phylogenetic investigations based on large data sets have previously addressed taxon relationships within Syndermata, though many questions remain. There is consistent evidence for paraphyly of “Eurotatoria” (Monogononta + Bdelloidea) with respect to Acanthocephala [6], [10], [17], [44], illustrating a non-parsimonious evolution including gains and losses of complex morphological traits during syndermatan evolution [10]. However, the branching order within a putative monophylum comprising Bdelloidea, Seisonidea, and Acanthocephala (“Hemirotifera”) [35] has remained unresolved. The so-called Lemniscea hypothesis favors a grouping of Acanthocephala and Bdelloidea and refers to potentially synapomorphic proboscis/rostrum and lemnisci/epidermal intrusions in acanthocephalans and bdelloids (Fig. 1A) [45]. However, the homology of the implied synapomorphies was frequently called into question [40], [46]–[51]. The alternative sister group relationship of seisonids and acanthocephalans (Pararotatoria) was derived from – amongst others – the shared occurrence of epidermal fibre bundles and so-called dense bodies in the spermatozoa [22], [23]. Monophyletic Pararotatoria additionally received support from analysis of partial 18S rDNA data [26], but were not reproduced in further analyses of 74 morphological characters and 18S rRNA, 28S rRNA, Hist3, and COI data [35], leaving Hemirotifera as a trifurcation (Fig. 1B). This apparent lack of resolution was most probably due to the small amount of data for Seisonidea, for which only the 18S rRNA sequence and partial sequences of 28S rRNA, hsp82 and COI were available in public databases. Resolving the phylogenetic position of Seisonidea, however, is essential to infer the evolution of acanthocephalan endoparasitism, which could have evolved via an epizoic intermediate stage.

In the present study, we analyze the internal phylogeny of Syndermata on the basis of novel cDNA sequence data for four syndermatan species (Seisonidea: *Seison* spec., Bdelloidea: *Adineta vaga*, Monogononta: *Brachionus manjavacas*, Acanthocephala: *Paratenuisentis ambiguus*) and additional EST data for *Pomphorhynchus laevis* (Acanthocephala). Our tree reconstructions are based on the first phylogenomic data set for Seisonidea. Moreover, we included twice as many syndermatan species as any previous phylogenetic study in this part of the metazoan tree.

## Materials and Methods

### Collection of material, RNA extraction, cDNA-synthesis and EST sequencing

Specimens of *P. laevis* were extracted from the gut of barbels (*Barbus barbus*, Teleostei), collected in the Buech River (South of France) in summer 2006. The study was conducted according to relevant international guidelines regarding the care and welfare of fishes and did not involve species that were endangered or protected (The IUCN Red List of Threatened Species v. 2011.1; www.iucnredlist.org). The Buech River is a regulated ‘Natura 2000’ area (FR9301519) and the permission for collecting fishes was delivered by the Préfecture des Alpes-de-Haute-Provence (N°2006-1259). The fishes were quickly sacrificed using clove oil as anesthetic to minimize suffering. Total RNA was isolated from one frozen adult *P. laevis* specimen to construct a cDNA library (Bio S&T Inc.). Double-stranded (ds) cDNA was directionally cloned into a modified pBluescript vector and transformed into *E. coli* DH10B cells. 10,000 bacterial colonies were randomly picked for single-pass 5′-end Sanger sequencing (Génoscope, Centre National de Séquençage, France). After controlling for quality and vector contamination, 9,374 validated EST sequences were compared to sequences available in the protein and nucleotide GenBank databases (SwissProt, NT and NR) using BLAST [52]. Sequences providing BLAST-matches with e-values <10^−15^ (n = 571) were chosen for full-length cDNA sequencing.

Specimens of *Nebalia bipes*, the host of *Seison nebaliae* and *Paraseison* (formerly *Seison*) *annulatus,* were collected in the tidal flats at Roscoff, France. After decapitation of *N. bipes* and removal of the carapax, epizoic *Seison*/*Paraseison* specimens were collected by stirring them off the gills of their host. About 95% of the sampled specimens belonged to *Seison nebaliae*, the remaining fraction belonged to *Paraseison annulatus*. For reasons of simplicity, we herein refer to the pooled specimens as to the *Seison* spec. sample. Total RNA extraction was performed using the NucleoSpin® RNA XS Kit (Macherey-Nagel) with approximately 100 *Seison* individuals. Specimens of *P. ambiguus* were collected from European eel (*Anguilla anguilla*, Teleostei) in the river Weser near Gimte (Germany). Total RNA extraction was performed using the RNeasy® Mini Kit (Qiagen) with five individuals. For both, the *P. ambiguus* and *Seison* spec. sample, RNA quality and concentration was checked on a denaturing 1.2% agarose/formaldehyde gel. Synthesis of ds cDNA was performed using the MINT Universal Kit (Evrogen), applying protocol II and 25 PCR cycles for 2nd strand synthesis. Subsequently, ds cDNA was purified using the QiaQuick PCR Purification Kit (Qiagen). The quality of ds cDNA was checked on the BioAnalyzer 2100 (Agilent) using a DNA High Sensitivity Chip. ESTs were sequenced using a Roche 454 Genome Sequencer FLX instrument. Due to the limited number of *Seison* specimens and a consequently small amount of input material for this taxon, we performed two different amplification approaches prior to sequencing. The cDNA used in the first sequencing run was subjected to an amplification step using the GenomePlex® Whole Genome Amplification (WGA) Kit (Sigma-Aldrich) prior to library preparation. For the second sequencing run the cDNA was amplified using the primer binding sites on the adapter molecules of the MINT-Universal Kit (M1-Primer). Sequencing of *P. ambiguus* ESTs was performed in the Max Planck Institute for Plant Breeding Research in Berlin (www.mpipz.mpg.de), sequencing of *Seison* spec. ESTs was performed by GENterprise Genomics (www.genterprise.de).

For *A. vaga* and *B. manjavacas*, total RNA was isolated from clonal cultures using the RNAqueous Micro Kit (Ambion). *A. vaga* RNA was treated with Terminator 5′-Phosphate Dependent Exonuclease (Epicentre). Synthesis of cDNA with first strand primer 5′ CTA GAG GCC GAG GCG GCC GAT TTT TTT TTT TTT TTT TTT UVN 3′ made use of the template switching property of Superscript II (Invitrogen) to incorporate barcoded, biotinylated 5′ adapters that matched the “A” sequence primers used in 454 FLX pyrosequencing (5′ GCC TCC CTC GCG CCA TCA Gxx xxx GG, where xxxxx is CACTG for *B. manjavacas* and ATCAG for *A. vaga*). To prepare libraries for pryrosequencing (Roche 454 Genome Sequencer FLX), 3–5 µg cDNA was sheared using an Aeromist Nebulizer (Allied Healthcare Products) for 3–4 min at 50psi N2, and the biotinylated 5′ EST ends of the fragmented library were captured with Dynabeads M-280 Streptavidin (Invitrogen). The captured DNA was end repaired using a Quick Blunt Kit (New England BioLabs) and ligated to a modified 454 FLX “B” adapter (AAG CCT TGC CAG CCC GCT CAG T) following A-tailing with *Taq* polymerase.

### EST processing

EST processing and assembly were performed at the Centre for Integrative Bioinformatics Vienna (deep-phylogeny.org) for all 19 species. EST data of four species *Seison* spec., *P. ambiguus, A. vaga* and *B. manjavacas* were newly attained in our study. Novel data for *P. laevis* were combined with already published EST data of that species. For the remaining 14 species in the phylogenetic analysis, ESTs were collected from public sequence resources such as the NCBI Trace Archive, dbEST (NCBI) or Gene Index Project and subsequently assembled into contigs as previously described [4], [53].

Processing of EST data included quality clipping, vector and adapter removal, poly-A removal and repeat masking. To this end we combined the programs Cross_match version 0.990329 (www.phrap.org), Lucy [54], SEQCLEAN (http://compbio.dfci.harvard.edu/tgi/software/) and RepeatMasker version open-3.1.6 (http://www.repeatmasker.org), in an in-house data processing workflow, using the databases UniVec (http://www.ncbi.nlm.nih.gov/VecScreen/UniVec.html) and RepBase [55]. Assembly was performed with MIRA v3.0.3 [56] or TGICL [57]. The results of processing and assembly are summarized in Table S1. Raw sequence data have been deposited in the Sequence Read Archive (SRA) for *Seison* spec., *P. ambiguus*, *A. vaga*, and *B. manjavacas* (accession numbers PRJEB1659, PRJEB1598, SRR800763, and SRR801079, respectively). New EST data of *P. laevis* used for phylogenetic reconstructions were deposited at EMBL under the accession numbers FO680693 - FO681285.

### Data set compilation

The collection of datasets used in this study is summarized in Table 1. Further details are provided in Table S2. For ortholog search, we used a pre-defined set of 1,253 ortholog groups [58] (lophotrochozoa_hmmer3, downloaded from http://www.deep-phylogeny.org/hamstr/download/datasets/hmmer3/) comprising sequences from the following species: *Schistosoma mansoni, Lottia gigantea, Helobdella robusta, Capitella capitata, Caenorhabditis elegans, Daphnia pulex* and *Apis mellifera*. We then used HaMStR [58] to extend these ortholog groups with sequences from the remaining species to be studied. We could extend 1,180 ortholog groups by adding sequences from our EST data sets. The orthologs for each protein were aligned with MAFFT [59] and the alignment was post-processed with GBlocks [60] using low stringency parameters as pre-defined on the GBlocks Server webpage (http://molevol.cmima.csic.es). Subsequent concatenation resulted in a supermatrix of 19 taxa and 1,180 genes. To find a subset of genes suitable for phylogenetic reconstructions, we applied MARE v0.1-rc (MAtrix REduction) [61]. This tool uses quartet mapping with extended geometry mapping to assign information content to a protein sequence. Additionally, data availability is taken into account using matrix reduction (e.g., keeping only genes that are available for at least 4 taxa). Using parameters t = 2 and d = 1.0, we compiled a reduced matrix “mintax4” (410 genes; Table 1). We also compiled a more stringent data set by only keeping genes for which at least 8 species contributed sequence information (“mintax8”; 272 genes; Table 1). The datasets “mintax4” and “mintax8” contained 51 and 50 ribosomal protein (RP) sequences, respectively. Given the occasionally questioned usefulness of RP data for phylogenetic analyses (e.g., [5], [62]) we also compiled “non-RP” data sets by deleting all RP sequences, resulting in data sets “mintax4_noRPs” and “mintax8_noRPs” (Table 1). Based on the 410-gene matrix “mintax4”, we eventually compiled a “most purposive subset” (“MPS”; Table 1). Here we selected from “mintax4” only those genes that were represented by at least one sequence in each taxon needed to address the question of internal syndermatan relationships. More precisely, we assigned our species to one of the following five groups: Acanthocephala (n = 3), Seisonidea (n = 1), Bdelloidea (n = 2), Monogononta (n = 2) and non-syndermatans (n = 11). Again, a modified data set without RP sequences was compiled as well (“MPS_noRPs”; Table 1). To address potential long-branch attraction (LBA) phenomena, we furthermore compiled a data set including only slowly evolving genes from the “mintax4” data set (“mintax4_slow”; Table 1). Evolutionary rates of the genes were approximated as described by Ebersberger et al. [53]. In brief, we used RAxML with the LG +I +G +F model to infer for each gene a maximum likelihood tree using only the primer taxon sequences. The total branch lengths of the resulting trees were then used as a proxy for the evolutionary rate of the corresponding gene. “Fast evolving” genes with values exceeding the 75% quantile were then excluded (see Figure S1).

**Table 1:** Overview of data sets and performed phylogenetic analyses

Data set	# proteins	# aa	analyses*
mintax4	410	76,652	1A, 2A, 3B, 4C
mintax4_noRPs	359	68,645	1A, 2A, 3B, 4C
mintax8	272	49,091	1A, 2A, 3B, 4C
mintax8_noRPs	222	41,296	1A, 2A, 3B, 4C
MPS	101	16,496	1D, 2D, 3E
MPS_noRPs	54	9,350	1D, 2D, 3E
mintax4_slow	307	60,753	1A
mintax4_4Synd	410	76,652	1D
mintax4_4Synd-DS	410	32,341	1D

The phylogenomic data sets mintax4 and mintax8 comprise a broad range of ortholog protein sequences detected using HaMStR and selected using MARE (keeping only genes that are available for at least four or eight taxa, respectively). The most purposive subset (MPS) data set comprises a fraction of the ortholog proteins in the mintax4 data set, that were at least partially covered by at least one representative per syndermatan subgroup plus one non-syndermatan species. Three additional data sets were compiled by excluding ribosomal proteins from the former mentioned protein selections (mintax4_noRPs, mintax8_noRPs, and MPS_noRPs). To account for potential long-branch attraction (LBA) errors, a data set comprising rather slowly evolving genes out of the mintax4 data set was compiled (mintax4_slow). Data set mintax4 was also modified by keeping only one species per syndermatan subgroup (*_4Synd) and subsequently diminished by deleting “singletons” and “dingletons” (*-DS). For each data set, the numbers of protein sequences (“# proteins”) and amino acid positions (“# aa”) are indicated. Performed analyses are encoded by numbers for the used programs (1 =  RAxML, 2 =  TreeFinder, 3 =  MrBayes, 4 =  PhyloBayes) and letters for the used substitution models (A =  8 partitions, B =  7 partitions, C =  CAT, D =  LG+I+G+F, E =  rtREV+I+G+F). For details see text and Supporting Information.

In a complementary approach to reduce branch lengths, we deleted alignment positions suspected of causing long branches and, correspondingly, LBA artifacts. In detail, we removed two types of positions, (i) where at least one of the aligned sequences exhibited a unique amino acid (private character or “singleton”) and (ii) where at least one pair of sequences shared an amino acid that is different from the amino acid seen in all other sequences (“dingleton”). Gaps were not considered. The goal was to reduce the lengths of terminal branches representing fast evolving species and, hence, to avoid artifacts from potential LBA. Note that this data modification is rather conservative as true phylogenetic signal can also be erased. Thus, when reproducing results even under these strict conditions, a certain topology can be regarded as robust and not due to LBA. Prior to these alignment modifications we reduced the number of species within Syndermata to one species per syndermatan subgroup, thus avoiding potential effects from unequal subgroup sampling (data set names with extension “4Synd”). For each syndermatan subgroup, we kept the taxon with the highest data coverage in the “mintax4” data set (Table S2).

### Determining substitution models and partitions

Best-fitting substitution models for the individual data were determined using ProtTest 2.4 [63] and the Akaike information criterion (AIC) [64]. For the concatenated “MPS” and “MPS_noRPs” data sets, LG [65] and rtREV [66], were determined as the first and second best fitting models of protein evolution, both with modelling invariant sites (option *I*), gamma distributed rate across sites (option *G*) and empirical base frequencies (option *F*). For the data sets with altered taxon sampling and those diminished by deleting “singletons” and “dingletons”, the LG model +*I* +*G* +*F* was selected. For the larger data sets (“mintax4”, “mintax8”, “mintax4_noRPs”, “mintax8_noRPs”, “mintax4_slow”), the best fitting evolutionary models were determined for each single protein alignment. Subsequently, the two best substitution models were extracted for every protein. Depending on their best-fit model the proteins were then assigned to eight different partitions (Blosum62, cpREV, Dayhoff, JTT, LG, rtREV, VT, WAG). Since the LG model was not implemented in MrBayes, the second best-fit models were used for the respective proteins. Hence the data set was subdivided into seven partitions (Blosum62, cpREV, Dayhoff, JTT, rtREV, VT, WAG) for MrBayes analysis. Options *I*, *G*, and *F* were used for all partitions in all subsequent tree reconstructions. For an overview of assigned substitution models and partitions see Table S3.

### Phylogenetic analyses

For tree reconstructions, we employed two maximum likelihood based programs (Treefinder [67], RAxML 7.2.8 [68]) and two Bayesian approaches (MrBayes 3.2.1 [69], [70], PhyloBayes 3.2b [71]). Statistical branch support was assessed by rapid bootstrapping [72] in RAxML (100 replicates), by LR-ELW edge support in TreeFinder (local rearrangement expected likelihood weights, approximate bootstrapping; 1,000 replicates) and by the Bayesian posterior probabilities.

For MrBayes analyses, we sampled every 100th out of a total of 1,000,000 generations for data sets <20,000 positions and every 10th out of 100,000 generations for data sets comprising >20,000 positions. Two parallel runs were carried out for all data sets, and we tested for convergence of the results by assessing the standard deviation of the split frequencies (StdDev). StdDev was <0.01 for all analyses except mintax4 (StdDev  = 0.02) and mintax8 (StdDev  = 0.01). In the MrBayes analyses of the partitioned data sets, all parameters (statefreq, revmat, shape, pinvar) were separately determined for each partition. Overall rate variation was allowed to be different across partitions by setting the ratepr parameter to variable. To allow for burn-in of the tree search, we discarded the first 25% of sampled trees in each analysis.

PhyloBayes analyses employing the CAT model were performed running two or three independent runs, each for a minimum of 16,000 generations. After a pairwise check for convergence using bpcomp from the PhyloBayes package, the consensus tree was built from the two runs with the smallest discrepancy observed across all bipartitions, discarding the first 1,000 trees as burn-in and sampling every second tree.

## Results

### Broad-scale reconstruction of syndermatan phylogeny

Initially, two different phylogenomic data sets were compiled for the 19 species sampled, containing proteins that were represented by orthologs of at least four or eight species, respectively. These phylogenomic data sets “mintax4” (410 genes, 76,652 positions) and “mintax8” (272 genes spanning 49,091 positions) included sequences of 51 and 50 RPs, respectively (equal to 10–20% of the amino acid positions). The suitability of RPs for phylogenetic reconstructions was lately called into question because of the suspicion of introducing a bias by “relying on a set of markers belonging to a single class of macromolecular complexes” [62] or by a compositional heterogeneity (i.e. deviating amino acid compositions) of RPs for certain taxa [5]. Therefore, two additional data sets were compiled by excluding RP sequences. Using these data sets we investigated whether RP sequences within the larger phylogenomic supermatrices were capable of influencing the resulting tree topologies or statistical support values. The resulting data sets comprised protein sequences for 359 genes (68,645 positions; “mintax4_noRPs”) and 222 genes (41,296 positions; “mintax8_noRPs”), respectively (Table 1). In a third approach we compiled a data set solely composed of proteins available for all taxa, which were central to the most important phylogenetic question at hand (i.e. the relationships of syndermatan subgroups). We thus split the taxa into five groups (Seisonidea, Bdelloidea, Monogononta, Acanthocephala, and non-syndermatans) and identified genes out of the mintax4 data set that were available for at least one member of each group. These genes were compiled into two data sets: a “most purposive subset” (MPS) data set (101 genes, 16,496 amino acid positions) and a “MPS_noRPs” data set lacking all RP sequences (54 genes, 9,350 amino acid positions).

**Figure 2:**
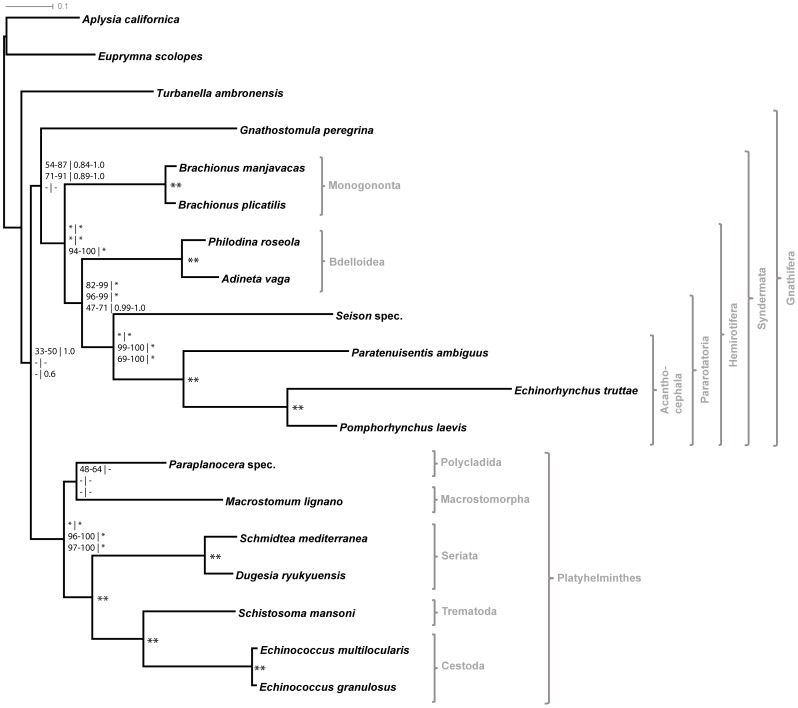
Phylogenetic relationships obtained with different phylogenomic data sets. Tree and branch lengths represent results of the TreeFinder analysis of the partitioned mintax4 data set. Node labels represent minimum and maximum support values of analyses of the phylogenomic data sets (mintax4, mintax8; upper row), phylogenomic data sets without RPs (mintax4_noRPs, mintax8_noRPs; middle row), and most purposive subset (MPS) data sets (with or without RPs; lower row). For further details see Table S4. ML bootstrap and ELW support (RAxML, TreeFinder) | PP values (MrBayes, PhyloBayes). A star denotes maximum support, a double star denotes maximum support in all analyses.

Regardless of the data set and tree reconstruction method used, the consensus trees supported the same syndermatan topology (Fig. 2, Table S4). For the data sets mintax4 and mintax8 (with or without RP sequences included), statistical support strongly suggested a sister group relationship of Seisonidea and Acanthocephala ( =  Pararotatoria; ML bootstrap/ELW support 99–100%; Bayesian posterior probability 100%; Fig. 2). The analyses further supported paraphyletic Eurotatoria, with Bdelloidea as the sister group of Pararotatoria ( =  Hemirotifera; Fig. 2). While support values for Hemirotifera were high using the mintax4 and mintax8 data sets (with or without RP sequences included; ML bootstrap/ELW support 82–99%; Bayesian posterior probability 100%; Fig. 2), monophyletic Gnathifera (Syndermata + Gnathostomulida) were recovered with only moderate support (ML bootstrap/ELW support 54–91%; Bayesian posterior probability 84–100%; Fig. 2). Analyses of the MPS data sets (with our without RP sequences included) provided weaker support for Pararotatoria (ML bootstrap/ELW support 69–100%; Bayesian posterior probability 100%; Fig. 2) and Hemirotifera (ML bootstrap/ELW support 47–71%; Bayesian posterior probability 99–100%; Fig. 2) and did not produce monophyletic Gnathifera. Finally, the phylogenetic position of the single gastrotrich species in our analyses, *T. ambronensis*, varied throughout the different tree reconstructions (Table S4). The same was observed for the branching order at the base of the Platyhelminthes clade.

### Focusing on slowly evolving proteins and addressing potential LBA

**Figure 3:**
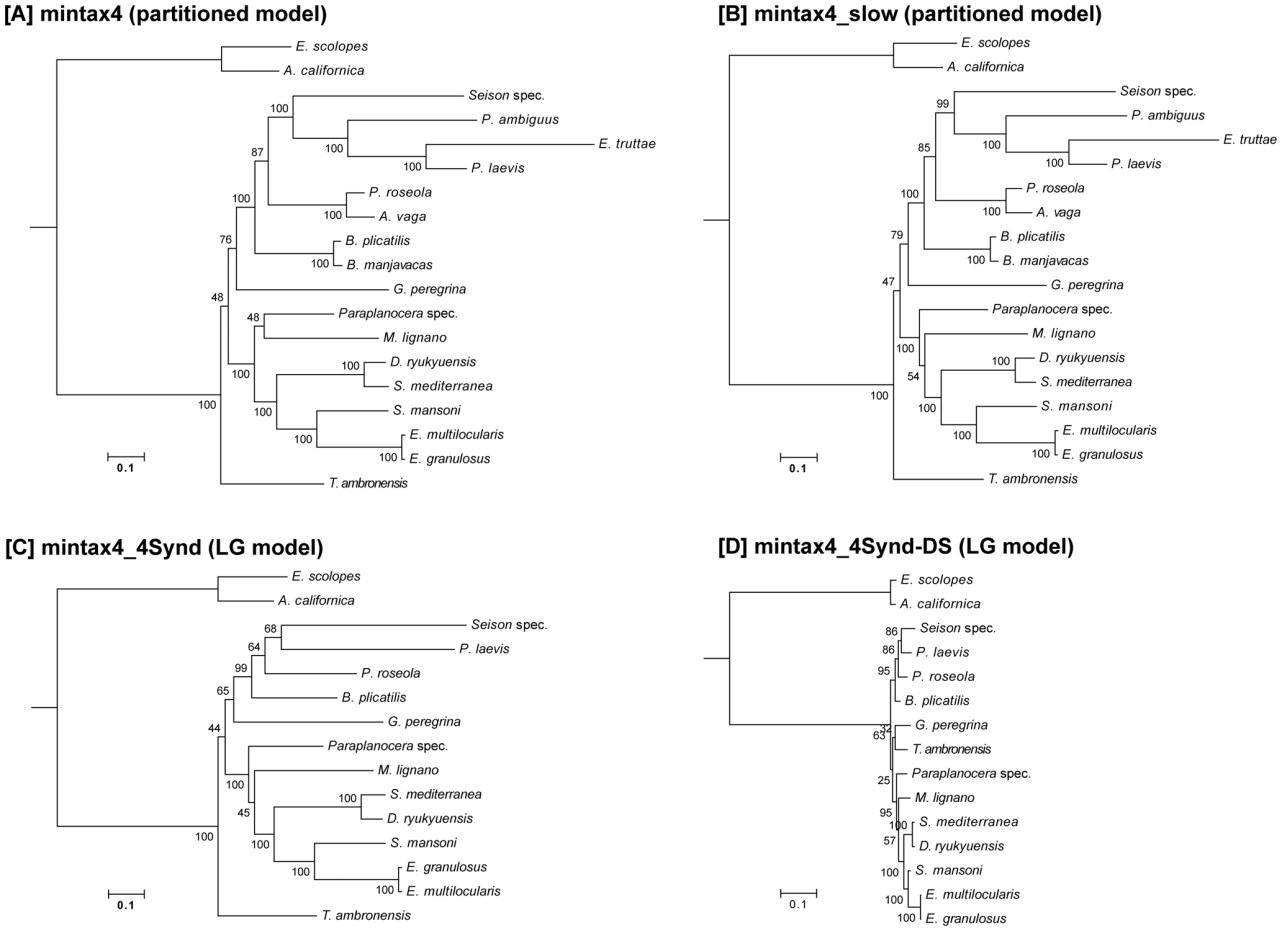
Comparison of branch lengths obtained with different data sets. Results of RAxML analyses based on [A] mintax4 data set, [B] mintax4_slow data set, [C] mintax4 data set with reduced taxon sampling within Syndermata (mintax4_4Synd), and [D] mintax4_4Synd data set excluding “singletons” and “dingletons” (mintax4_4Synd-DS).

To assess how the exclusion of fast-evolving genes influences our phylogenetical conclusions, we removed the 25% fastest evolving genes from mintax4, resulting in dataset “mintax4_slow” (Figure S1). Notably, we observed the same syndermatan topology and almost identical support values for Pararotatoria, Hemirotifera, Syndermata and Gnathifera (Fig. 3A and B).

To further account for potential LBA errors in our tree reconstructions, we conducted additional RAxML analyses on a modified mintax4 data set containing only 4 syndermatan species (data set mintax4_4Synd). This was done to create a balanced representation of the 4 syndermatan subgroups, concomitantly maximizing sequence coverage. We then pruned from this alignment all columns containing private characters, unique to one species (“singletons”), and also those where sequence pairs shared a deviating character state (“dingletons”). This was intended to remove sites contributing to long branches and their potential for attracting each other by convergent substitutions. The resulting data set was named mintax4_4Synd-DS. Both approaches (mintax4_4Synd, mintax4_4Synd-DS) recovered the well-supported syndermatan relationships previously seen in the standard analyses (see Fig. 3B and D; see also Table S4). Note, that decreasing taxon sampling within Syndermata (mintax4_4Synd) substantially reduced bootstrap support for nodes of the internal syndermatan topology (Fig. 3C). Interestingly, this effect was counterbalanced by the deletion of “singletons” and “dingletons” (-DS), which resulted in substantially reduced branch lengths, and re-established bootstrap support values >85 for the internal nodes (Fig. 3D). Taken the results of all datasets together, we consistently observed monophyletic Pararotatoria, Hemirotifera, and Syndermata irrespective of the diverse alignment modifications and tree reconstruction methods used.

### Phylogenetic relations beyond Syndermata

Our data was particularly designed for resolving syndermatan phylogeny. Still it is noteworthy, that monophyletic Gnathifera could only be observed with moderate support in all phylogenetic analyses except for those based on the MPS, MPS_noRPs and mintax4_4Synd-DS datasets (Table S4). The position of the gastrotrich species varied throughout the analyses, without affecting syndermatan topology. Finally, evolutionary relationships within Platyhelminthes were stable throughout the analyses and in most parts maximally supported (Figs. 2 and 3). The topology of the platyhelminth subtree agreed with the generally accepted monophyletic origin of the parasitic flatworm groups (i.e. Cestoda, Trematoda and Monogenea; summarized as Neodermata) and a paraphyletic status of free-living “Turbellaria” (see [73] for a detailed discussion about proposed hypotheses). Seriata appeared as sister group to Neodermata in our trees, whereas the other “turbellarian” species (*Paraplanocera* spec. and *Macrostomum lignano*) split off at the base of the flatworm clade. The only inconsistency within the flatworm clade among our analyses proved to be the variable branching pattern of these two flatworm species (see Table S4).

## Discussion

### Syndermatan phylogeny and implications for the evolution of parasitism

**Figure 4:**
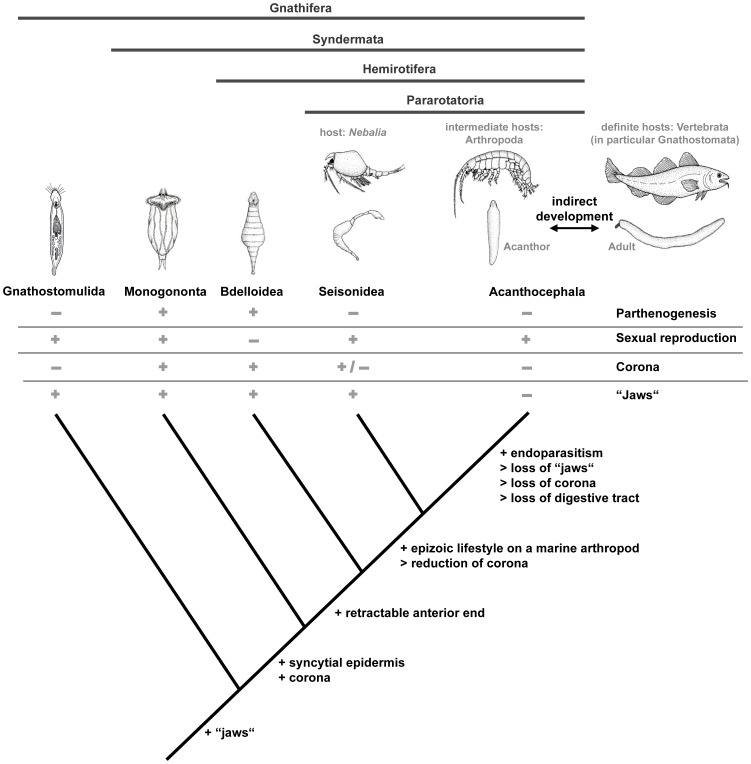
Implications for character evolution in Syndermata. Morphological and biological features of representatives of Syndermata and Gnathostomulida were projected on our phylogenetic tree (see Figs. 2 and 3). Present features are denoted with a plus, absent features are denoted with a minus. The corona in Seisonidea is reduced (denoted with plus/minus). The pictogram of Gnathostomulida has been modified from biodidac.bio.uottawa.ca. Pictograms of other representatives: courtesy of Bernd Baumgart (Göttingen, Germany).

We have conducted the to-date most comprehensive phylogenomic analysis of syndermatan relationships, for the first time including large-scale molecular data for Seisonidea, and hence for all higher-ranked clades of this animal group. Tree reconstructions on 9 different phylogenomic data sets using 4 different tree reconstruction methods reveal a consistent view on syndermatan phylogeny. The analyses provide strong support for the monophyly of Syndermata (Monogononta + Hemirotifera), Hemirotifera (Bdelloidea + Pararotatoria) and Pararotatoria (Seisonidea + Acanthocephala). Our results thus contradict previous hypotheses of monophyletic Eurotatoria (Bdelloidea + Monogononta) and Lemniscea (Acanthocephala + Bdelloidea). Mapping lifestyles on the newly inferred tree topology, the most parsimonious evolutionary scenario is the following (Fig. 4): the obligate endoparasitism of Acanthocephala evolved from free-living ancestors via an epizoic, possibly even ectoparasitic stage as represented by extant species of Seisonidea. A similar scenario has also been hypothesized for the evolution of parasitism within neodermatan Platyhelminthes [74]–[76], where the vast majority of Trematoda and Cestoda are endoparasites while Monogenea exhibit an ectoparasitic lifestyle. Since there is growing molecular evidence for a paraphyletic status of Monogenea [75]–[78], the most probable scenario for the evolution of parasitism within this flatworm clade is an ectoparasitic neodermatan progenitor and subsequent shifts towards endoparasitism on the trematode/cestode branch [75], [76].

Considering the host spectra of Seisonidea and Acanthocephala and assuming that the situation in Seisonidea represents the ancestral state (see Fig. 4), marine arthropods probably were the hosts of early acanthocephalans. These initial hosts then became intermediate hosts, following the introduction of marine fish-like gnathostomes or vertebrates as additional definitive hosts [26], [79], [80]. With the diversifying evolution of the host-species, acanthocephalans established a broadened host spectrum and extended their distribution to limnic and terrestrial habitats [26], [79], [80]. The successive recruitment of arthropods and gnathostomes/vertebrates as acanthocephalan hosts is in line with principal considerations regarding the evolution of increasingly complex life-cycles, whereupon vertebrates fed on potentially infected arthropods after one-host life cycles were established [81]. Moreover, this scenario implies that the indirect development of acanthocephalans, involving the metamorphosis from a larval stage (acanthor) to a young adult (acanthella; called cystacanth when encysted) within the intermediate host [82], represents an evolutionary novelty of acanthocephalans. It is obvious that morphological traits such as the rostellar apparatus of the acanthor, a proboscis including hooks and specific musculature of infectious acanthella and adults, and a reduced digestive tract throughout all developmental stages are acanthocephalan autapomorphies that most probably evolved in the context of their endoparasitic lifestyle [18], [22], [50], [79], [83] (Fig. 4). We would like to point out that presumed morphological peculiarities of acanthocephalans such as anastomoses between the epidermal rete system and hollow muscle fibres [84] were not observed in follow-up investigations of praesomata of different acanthocephalan species [43] and may therefore not represent a genuine morphological character of acanthocephalans.

Our phylogenetic tree is in line with the proposed ultrastructural autapomorphies of Pararotatoria, i.e. epidermal fibre bundles and dense bodies in the spermatozoa [22], [23]. We further suggest that the last common ancestor of Pararotatoria reproduced strictly sexually, whereas monogononts and bdelloids retained or independently evolved the ability for parthenogenesis (for a more detailed view on character distribution, see Table 13.1 in [85]). The last common ancestor of Pararotatoria may further have had a reduced rotatory organ or corona. This can easily be explained by a reduction of the character after its emergence in the stem lineage of Syndermata [10] (Fig. 4). However, the rotatory or wheel-organ is not the only example of a putative emergence and subsequent loss of complex morphological traits in syndermatan evolution. Weber et al. [44] mapped the occurrence of apical and lateral sense organs in acanthocephalans onto a tree topology obtained from complete mitochondrial genome data and revealed a “come and go” distribution among the acanthocephalan lineages. Finally, a retractable anterior end, whether called rostrum or proboscis, might represent an evolutionary novelty of Hemirotifera [10] (Fig. 4).

### Influence of long branches on syndermatan phylogeny

In molecular phylogeny, syndermatan taxa are well known to display long branches, probably caused by fast sequence evolution, which might lead to erroneous tree topologies due to the LBA artifact (e.g., [2], [78], [86]). A substantial reduction of branch lengths and therefore of the potential impact of LBA could be observed when removing “singletons” and “dingletons” from the data set (Fig. 3D). However, deleting those alignment sites not only shortens branch lengths, but also potentially removes positions containing true phylogenetic signal. Despite this possibility, we observed that this approach resulted in substantially increased support values for Pararotatoria and Hemirotifera compared to the tree based on the mintax4_4Synd data set (Fig. 3C and D). In summary, the robustness of the branching order within Syndermata irrespective of taxon composition and data set modifications (Figs. 2 and 3, Table S4) lends strong support to the assumption that our tree topology is most likely not heavily compromised by LBA artifacts.

### Towards understanding deeper phylogenetic relationships of Syndermata

Together with other small animal taxa (Gnathostomulida, Micrognathozoa and, possibly, Cycliophora), Syndermata hypothetically form the superordinate taxon Gnathifera, which is characterized by pharyngeal hard parts (“jaws”) with particular supportive rods as an eponymous evolutionary novelty (e.g. [22], [87], [88]; see also [89]). Monophyly of Gnathifera was also obtained in molecular phylogenetic studies [2], [6], [31]. In particular, RP sequence data revealed moderate to good statistical support for monophyletic Gnathifera [2], [6]. However, depending on the applied evolutionary model and most probably due to LBA, Gnathifera artificially emerged as part of Ecdysozoa in the study of Hausdorf et al. [2]. Conceivably, such LBA phenomena could have been caused by co-evolution of RPs with rapidly evolving rRNA sequences [62]. Other multi-gene and phylogenomic analyses depicted the few sampled gnathiferan species as unstable and did not support monophyletic Gnathifera [1], [11], [12]. In the present study, some data sets produced moderate support for monophyletic Gnathifera, while others did not (ML bootstrap/ELW support 54–91%; Bayesian posterior probability 84–100%; see Figs. 2 and 3 and Table S4). Thus, LBA might have contributed to previous reports of monophyletic Gnathifera in molecular-phylogenetic reconstructions. Alternatively, the occasional observation of non-monophyletic Gnathifera in the present study (gnathostomulids appeared as sister to gastrotrichs in most of these cases, see Table S4) might be due to the extremely low data coverage of the single gastrotrich taxon, *T. ambronensis,* which amounted to only 8.7% and 13.3% in the phylogenomic data sets mintax4/8 (Table S2). It is well known that such low data coverage, especially in combination with the absence of closely related taxa (here: other gastrotrichs) in the data set, can potentially affect accuracy of molecular phylogenies [14]. This issue, and whether other taxa such as Micrognathozoa and Cycliophora belong to putative Gnathifera as well, will be the subject of future research. This also pertains to the affiliations of putative Gnathifera within the metazoan tree, e.g. the hypothetical super-taxon Platyzoa [9].

The example of syndermatan phylogeny shows that improvements in taxon and data coverage, along with the approach to compile different data sets with variable properties, can lead to stable and reliable solutions for phylogenetic questions, even if long branches are involved. The addition of the species *P. ambiguus* and *Seison* spec. efficiently disrupted the long branch leading to Acanthocephala and made tree reconstructions more reliable compared to previous studies. Also, support values were higher the more syndermatan species were involved (compare Fig. 3A and C). Increasing transcriptome data availability for syndermatan species thus is an initial step towards resolving the Gnathifera and Platyzoa questions. Improved data collection within Gastrotricha and Gnathostomulida has been initiated and will facilitate to reasonably address these deep phylogenetic hypotheses.

## Supporting Information

Figure S1 **Evolutionary rates of the HaMStR core orthologs and the orthologs contained in the datasets.** Box plots depict the median, minimum and maximum evolutionary rates (as calculated from tree lengths) for the gene orthologs, which make up the individual phylogenomic datasets. For definition of these datasets, see Material and Methods.
(PDF)

Table S1 **Results of EST processing and assembly.** For all newly sequenced species, the applied sequencing method, total number of sequencing reads, number of reads discarded during preprocessing and total number of cleaned ESTs are given. Reads were discarded during pre-processing if they (i) did not exceed the minimum length of 100 bp after adapter and quality trimming, (ii) contained more than 3% of undetermined bases, (iii) were mainly of low complexity, or (iv) other reasons. The cleaned ESTs were assembled using MIRA v3.0.3 or TGICL, the number of contigs, number of single reads as well as the length of the largest contig and N50 size of the assembly are denoted. SC  =  standard chemistry, TC  =  Titanium chemistry, n.d.  =  not determined.
(PDF)

Table S2 **Dataset coverage of single taxa (% amino acid positions, number of proteins).** For the different species used in our study, the values display the percentage to which extent these taxa are covered in terms of amino acid positions and number of proteins used in the concatenated alignments of the phylogenomic datasets. These datasets are further specified in Material and Methods.
(PDF)

Table S3 **Assignment of substitution models and composition of the phylogenomic datasets.** The matrix specifies (i) which ortholog (listed by its ID) is present in which of the phylogenomic datasets and (ii) the best and the second-best substitution model for calculating a respective phylogenetic tree. Ribosomal proteins are denoted by their short designations (e.g. S18) and their corresponding Caenorhabditis elegans gene name. The phylogenomic datasets are further specified in Material and Methods.
(PDF)

Table S4 **Support values for internal nodes, position of Turbanella and position of Paraplanocera spec. and M. lignano obtained by all analyses.** Statistical support values as obtained from four different phylogenetic reconstruction programs are given for the phylogenomic datasets, defined in detail in Material and Methods. Taxon abbreviations are: S =  *Seison*, A =  Acanthocephala, B =  Bdelloidea, M =  Monogononta, Syn  =  Syndermata, G =  Gnathostomulida, T =  Turbanella ambronensis. (*) Possible phylogenetic positions for the gastrotrich *Turbanella ambronensis* ( = "*Turbanella* ") are: 1 =  sister to (Platyhelminthes + Syndermata + Gnathostomulida), 2 =  sister to Gnathifera, 3 =  trichotomy (Mollusca,*Turbanella*,(Platyhelminthes+Gnathifera)), 4 =  sister to Platyhelminthes, 5 =  clade (*Turbanella* + *Gnathostomula*) sister to Syndermata, 6 =  sister to Syndermata, 7 =  clade (*Turbanella* + *Gnathostomula*) sister to Platyhelminthes.
(PDF)
