# Supplementary material for: Transcriptome Data Reveal Syndermatan Relationships and Suggest the Evolution of Endoparasitism in Acanthocephala via an Epizoic Stage
Source: PLoS One. 2014 Feb 10;9(2):e88618. doi: 10.1371/journal.pone.0088618 (PMC3919803; doi:10.1371/journal.pone.0088618)

Figure S1 - Evolutionary rates of the HaMStR core orthologs and the orthologs contained in the datasets

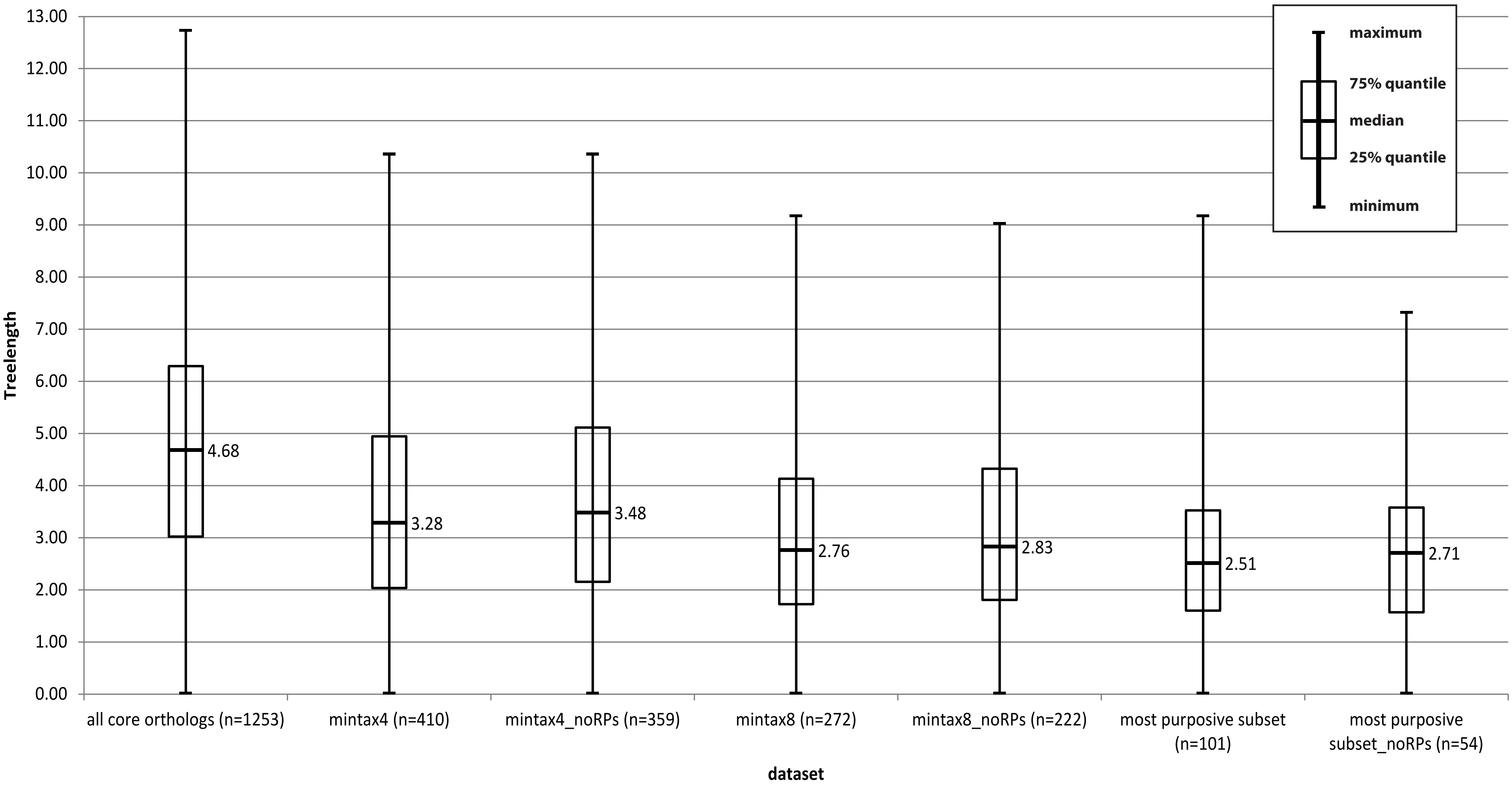

Supplement: Figure S1 — Evolutionary rates of the HaMStR core orthologs and the orthologs contained in the datasets. Box plots depict the median, minimum and maximum evolutionary rates (as calculated from tree lengths) for the gene orthologs, which make up the individual phylogenomic datasets. For definition of these datasets, see Material and Methods. (PDF) [file pone.0088618.s001.pdf]
