# Supplementary material for: Transcriptome Data Reveal Syndermatan Relationships and Suggest the Evolution of Endoparasitism in Acanthocephala via an Epizoic Stage
Source: PLoS One. 2014 Feb 10;9(2):e88618. doi: 10.1371/journal.pone.0088618 (PMC3919803; doi:10.1371/journal.pone.0088618)
Supplement: Table S1 — Results of EST processing and assembly. For all newly sequenced species, the applied sequencing method, total number of sequencing reads, number of reads discarded during preprocessing and total number of cleaned ESTs are given. Reads were discarded during pre-processing if they (i) did not exceed the minimum length of 100 bp after adapter and quality trimming, (ii) contained more than 3% of undetermined bases, (iii) were mainly of low complexity, or (iv) other reasons. The cleaned ESTs were assembled using MIRA v3.0.3 or TGICL, the number of contigs, number of single reads as well as the length of the largest contig and N50 size of the assembly are denoted. SC = standard chemistry, TC = Titanium chemistry, n.d. = not determined. (PDF) [file pone.0088618.s002.pdf]

Table S1 – Results of EST processing and assembly

For all newly sequenced species, the applied sequencing method, total number of sequencing reads, number of reads discarded during pre-processing and total number of cleaned ESTs are given. Reads were discarded during pre-processing if they (i) did not exceed the minimum length of 100 bp after adapter and quality trimming, (ii) contained more than 3% of undetermined bases, (iii) were mainly of low complexity, or (iv) other reasons. The cleaned ESTs were assembled using MIRA v3.0.3 or TGICL, the number of contigs, number of single reads as well as the length of the largest contig and N50 size of the assembly are denoted. SC = standard chemistry, TC = Titanium chemistry, n.d. = not determined.

			processing		assembly				
**species**	**sequencing method**	**sequencing reads**	**discarded during pre-processing**	**cleaned ESTs**	**used program**	**contigs**	**single reads**	**largest contig [bp]**	**N50 [bp]**
Seison spec.	454 (SC)	120,822	5,668	115,154	MIRA	7,682	3,142	2,1	483
P. ambiguus	454 (TC)	101,387	4,31	97,077	MIRA	8,264	1,561	1,801	404
A. vaga	454 (TC)	576,456	36,941	539,515	MIRA	15,578	3,388	2,1	462
B. manjavacas	454 (SC)	108,705	20,755	119,512	TGICL	11,629	n.d.	2,102	259
	Sanger	43,584	12,022						
