# Supplementary material for: Transcriptome Data Reveal Syndermatan Relationships and Suggest the Evolution of Endoparasitism in Acanthocephala via an Epizoic Stage
Source: PLoS One. 2014 Feb 10;9(2):e88618. doi: 10.1371/journal.pone.0088618 (PMC3919803; doi:10.1371/journal.pone.0088618)
Supplement: Table S2 — Dataset coverage of single taxa (% amino acid positions, number of proteins). For the different species used in our study, the values display the percentage to which extent these taxa are covered in terms of amino acid positions and number of proteins used in the concatenated alignments of the phylogenomic datasets. These datasets are further specified in Material and Methods. (PDF) [file pone.0088618.s003.pdf]

Table S2 - Dataset coverage of single taxa (%amino acid positions, number of proteins)

100%	0%					
**Taxon**	**mintax4**	**mintax8**	**mintax4 _noRPs**	**mintax8 _noRPs**	**most purposive subset**	**most purposive subset_noRPs**
Adineta vaga	20,11	29,08	13,04	18,92	58,68	39,42
Aplysia californica	65,85	76,26	61,99	71,98	85,01	74,43
Brachionus manjavacas	48,73	61,15	43,65	54,99	72,40	55,59
Brachionus plicatilis	85,55	93,41	84,78	93,17	92,99	91,29
Dugesia ryukyuensis	50,86	62,58	49,44	62,17	64,79	61,75
Echinococcus granulosus	31,47	41,71	25,54	33,62	57,29	33,83
Echinococcus multilocularis	13,04	17,94	10,51	14,59	24,76	13,93
Echinorhynchus truttae	14,53	19,95	13,46	19,11	24,70	23,26
Euprymna scolopes	50,29	59,00	47,44	56,16	67,63	63,10
Gnathostomula peregrina	17,46	26,69	11,15	17,84	46,96	24,89
Macrostomum lignano	27,56	39,75	22,45	33,42	46,30	27,30
Paraplanocera spec.	21,12	32,82	14,08	23,22	57,53	33,21
Paratenuisentis ambiguus	38,35	53,04	32,75	46,32	70,48	55,48
Philodina roseola	25,85	38,79	17,79	27,70	82,04	70,18
Pomphorhynchus laevis	49,38	62,00	46,03	58,57	71,17	61,79
Schistosoma mansoni	98,75	98,75	98,73	98,72	98,93	98,99
Schmidtea mediterranea	86,44	91,78	86,36	92,21	91,49	88,26
Seison spec.	31,44	44,50	25,26	37,02	84,91	79,65
Turbanella ambronensis	8,67	13,26	2,98	4,64	31,48	7,86
**average**	**41,34**	**50,66**	**37,23**	**45,49**	**64,71**	**52,85**

100%	0%					
**Taxon**	**mintax4 (n=410)**	**mintax4 _noRPs (n=359)**	**mintax8 (n=272)**	**mintax8 _noRPs (n=222)**	**most purposive subset (n=101)**	**most purposive subset_noRPs (n=54)**
Adineta vaga	136	87	125	76	79	33
Aplysia californica	311	260	236	186	92	45
Brachionus manjavacas	270	220	217	168	89	43
Brachionus plicatilis	371	323	258	210	95	50
Dugesia ryukyuensis	218	183	174	139	65	31
Echinococcus granulosus	173	128	147	102	67	24
Echinococcus multilocularis	70	52	62	44	28	10
Echinorhynchus truttae	67	54	60	47	29	16
Euprymna scolopes	263	224	200	162	77	42
Gnathostomula peregrina	104	63	101	60	58	19
Macrostomum lignano	126	87	116	77	50	14
Paraplanocera spec.	101	58	100	57	61	19
Paratenuisentis ambiguus	242	192	212	162	93	46
Philodina roseola	128	78	122	72	89	42
Pomphorhynchus laevis	232	188	188	144	78	36
Schistosoma mansoni	410	359	272	222	101	54
Schmidtea mediterranea	360	313	252	205	95	49
Seison spec.	175	127	157	110	101	54
Turbanella ambronensis	59	23	58	22	43	8
**average**	**201**	**159**	**161**	**119**	**73**	**33**
