# Supplementary material for: Transcriptome Data Reveal Syndermatan Relationships and Suggest the Evolution of Endoparasitism in Acanthocephala via an Epizoic Stage
Source: PLoS One. 2014 Feb 10;9(2):e88618. doi: 10.1371/journal.pone.0088618 (PMC3919803; doi:10.1371/journal.pone.0088618)
Supplement: Table S3 — Assignment of substitution models and composition of the phylogenomic datasets. The matrix specifies (i) which ortholog (listed by its ID) is present in which of the phylogenomic datasets and (ii) the best and the second-best substitution model for calculating a respective phylogenetic tree. Ribosomal proteins are denoted by their short designations (e.g. S18) and their corresponding Caenorhabditis elegans gene name. The phylogenomic datasets are further specified in Material and Methods. (PDF) [file pone.0088618.s004.pdf]

Table S3 - Assignment of substitution models and composition of the phylogenomic datasets

mintax4	mintax4_slow	mintax8	most purposive subset	ribosomal protein	*C. elegans* gene name	corresponding RP	Ortholog ID	Best Model	second best model
X							111209	LG	RtREV
X	X						111212	LG	Blosum62
X	X	X					111214	LG	WAG
X	X	X					111215	LG	RtREV
X	X	X	X	X	rps-23	S23	111216	LG	RtREV
X	X	X	X	X	rps-13	S13	111217	LG	JTT
X	X	X					111222	LG	JTT
X							111225	RtREV	LG
X	X	X					111229	LG	JTT
X		X					111230	JTT	LG
X	X						111233	LG	RtREV
X	X	X					111237	LG	JTT
X	X	X					111238	RtREV	LG
X							111242	CpREV	WAG
X	X	X					111243	LG	JTT
X	X	X	X				111249	LG	WAG
X	X	X	X				111259	LG	WAG
X							111263	CpREV	Blosum62
X							111265	LG	RtREV
X	X	X					111266	LG	RtREV
X	X						111270	LG	RtREV
X	X	X	X	X	rps-17	S17	111274	LG	RtREV
X	X	X					111275	LG	JTT
X	X	X	X	X	rps-11	S11	111277	LG	RtREV
X	X	X					111278	LG	RtREV
X	X						111279	LG	RtREV
X	X	X					111280	LG	WAG
X		X					111285	LG	RtREV
X	X	X	X				111286	LG	WAG
X	X	X	X				111287	LG	WAG
X	X	X					111290	LG	JTT
X		X					111291	LG	RtREV
X	X	X					111295	LG	RtREV
X	X						111296	LG	JTT
X							111297	LG	Blosum62
X	X						111298	LG	JTT
X	X	X					111299	LG	WAG
X	X	X	X				111305	LG	CpREV
X	X	X					111309	LG	RtREV

mintax4	mintax4_slow	mintax8	most purposive subset	ribosomal protein	*C. elegans* gene name	corresponding RP	Ortholog ID	Best Model	second best model
X		X					111313	LG	WAG
X	X						111315	LG	JTT
X	X	X					111316	LG	RtREV
X		X	X	X	rpl-28	L28	111318	LG	WAG
X		X					111328	RtREV	LG
X							111331	LG	RtREV
X							111334	Blosum62	LG
X	X	X					111335	WAG	LG
X							111344	LG	RtREV
X	X	X					111345	LG	RtREV
X	X	X	X				111347	WAG	LG
X	X	X					111349	LG	WAG
X	X						111350	LG	RtREV
X	X						111352	LG	Blosum62
X	X						111356	LG	JTT
X	X						111357	Blosum62	LG
X	X	X					111367	LG	Blosum62
X							111376	LG	RtREV
X							111377	VT	Blosum62
X	X						111379	LG	JTT
X		X					111388	LG	CpREV
X	X	X					111393	LG	CpREV
X	X	X	X	X	rpl-11.2	L11	111394	LG	WAG
X							111395	LG	WAG
X	X	X					111399	LG	WAG
X	X	X	X				111405	LG	RtREV
X	X	X	X				111413	LG	RtREV
X	X	X					111414	LG	WAG
X							111419	WAG	LG
X	X	X					111421	LG	RtREV
X		X	X				111423	LG	RtREV
X	X						111425	VT	Blosum62
X	X	X					111426	LG	RtREV
X		X					111427	LG	RtREV
X		X	X				111429	LG	RtREV
X		X					111430	LG	WAG
X	X	X					111432	LG	RtREV
X	X	X	X				111435	LG	WAG
X		X					111436	RtREV	LG
X	X						111444	LG	RtREV
X	X						111448	LG	Blosum62
X	X	X	X				111451	LG	RtREV

mintax4	mintax4_slow	mintax8	most purposive subset	ribosomal protein	*C. elegans* gene name	corresponding RP	Ortholog ID	Best Model	second best model
X	X	X	X				111457	JTT	LG
X	X						111459	RtREV	LG
X	X						111463	LG	WAG
X	X	X	X	X	rps-18	S18	111466	LG	RtREV
X	X	X					111469	LG	RtREV
X							111471	JTT	LG
X	X	X					111475	LG	RtREV
X							111481	LG	RtREV
X							111483	LG	VT
X	X	X	X				111485	LG	RtREV
X	X	X	X	X	rpl-13	L13	111491	LG	RtREV
X							111493	LG	WAG
X	X	X	X				111496	LG	RtREV
X	X	X					111497	LG	RtREV
X	X						111501	LG	RtREV
X	X	X					111504	LG	RtREV
X	X						111507	LG	Blosum62
X							111509	LG	CpREV
X	X	X	X				111510	LG	RtREV
X	X	X					111516	LG	RtREV
X							111518	LG	RtREV
X	X	X	X				111519	LG	RtREV
X		X					111521	LG	RtREV
X		X					111525	LG	JTT
X	X	X	X				111527	LG	JTT
X	X	X	X				111532	LG	CpREV
X							111535	CpREV	LG
X	X	X	X				111538	Blosum62	RtREV
X	X	X					111539	LG	RtREV
X	X	X					111542	LG	RtREV
X		X					111544	LG	JTT
X	X	X	X	X	rpl-21	L21	111546	LG	RtREV
X	X						111553	LG	RtREV
X	X	X	X	X	rpl-43	L37A	111555	LG	RtREV
X	X						111558	LG	RtREV
X	X						111560	LG	RtREV
X	X						111561	LG	WAG
X	X	X	X				111566	LG	RtREV
X	X	X					111568	LG	WAG
X							111570	LG	RtREV
X	X	X					111572	LG	RtREV
X		X					111576	JTT	LG

mintax4	mintax4_slow	mintax8	most purposive subset	ribosomal protein	*C. elegans* gene name	corresponding RP	Ortholog ID	Best Model	second best model
X	X						111579	LG	JTT
X							111583	LG	RtREV
X	X	X	X				111585	LG	WAG
X	X	X	X	X	rps-7	S7	111587	LG	RtREV
X							111588	JTT	LG
X	X	X	X	X	rps-9	S9	111595	LG	JTT
X		X					111596	LG	WAG
X	X	X					111598	LG	RtREV
X	X	X		X	rpl-23	L23	111599	RtREV	LG
X		X					111600	LG	Blosum62
X	X	X					111603	LG	Dayhoff
X	X	X					111604	LG	Dayhoff
X	X						111607	LG	RtREV
X	X	X	X				111608	VT	LG
X							111611	LG	RtREV
X	X	X					111618	LG	WAG
X	X						111619	LG	WAG
X		X					111620	LG	RtREV
X	X	X	X	X	rpl-22	L22	111622	LG	WAG
X	X	X					111623	LG	WAG
X	X	X					111625	JTT	LG
X	X	X	X				111626	LG	RtREV
X		X					111628	LG	WAG
X	X	X					111629	LG	WAG
X	X	X					111631	LG	RtREV
X	X						111634	LG	RtREV
X	X	X					111636	LG	RtREV
X		X					111639	RtREV	LG
X	X	X					111643	LG	JTT
X	X						111647	LG	RtREV
X	X	X	X				111648	LG	RtREV
X		X					111653	LG	CpREV
X							111654	WAG	LG
X	X	X	X				111657	LG	RtREV
X	X	X					111661	LG	Dayhoff
X	X	X					111668	LG	JTT
X	X	X					111676	CpREV	LG
X	X	X					111677	JTT	WAG
X	X	X	X				111678	LG	WAG
X							111679	LG	Blosum62
X	X	X					111681	WAG	Dayhoff
X	X	X					111686	LG	JTT

mintax4	mintax4_slow	mintax8	most purposive subset	ribosomal protein	*C. elegans* gene name	corresponding RP	Ortholog ID	Best Model	second best model
X		X					111699	VT	LG
X	X						111701	LG	RtREV
X	X	X	X	X	rpl-35	L35	111702	LG	RtREV
X	X	X					111707	LG	CpREV
X	X	X	X	X	rpl-24.1	L24	111711	LG	RtREV
X	X	X					111723	LG	JTT
X							111729	LG	WAG
X	X	X	X				111730	LG	JTT
X	X	X					111731	LG	RtREV
X	X	X	X	X	rpl-31	L31	111733	LG	RtREV
X	X	X					111734	WAG	LG
X	X	X					111738	RtREV	LG
X	X	X					111741	LG	JTT
X	X	X	X	X	rpl-10	L10	111744	LG	RtREV
X							111747	LG	RtREV
X	X						111749	LG	WAG
X	X	X					111763	LG	WAG
X	X	X					111768	LG	RtREV
X							111770	LG	Blosum62
X	X	X	X	X	rpl-4	L4	111775	LG	RtREV
X	X						111776	JTT	WAG
X	X						111777	LG	WAG
X	X						111778	LG	RtREV
X	X	X					111781	RtREV	LG
X							111784	LG	RtREV
X	X						111786	LG	RtREV
X	X	X					111787	LG	RtREV
X	X	X					111789	JTT	Dayhoff
X							111792	LG	JTT
X							111795	LG	WAG
X	X	X					111796	LG	RtREV
X	X	X					111797	LG	RtREV
X	X	X					111803	LG	RtREV
X	X						111807	LG	WAG
X	X	X					111812	LG	Blosum62
X	X	X	X	X	rpl-17	L17	111814	LG	JTT
X	X	X	X	X	rps-22	S15A	111815	LG	RtREV
X	X						111816	Dayhoff	DCMut
X	X						111818	LG	JTT
X	X						111820	LG	RtREV
X							111825	LG	WAG
X							111828	LG	RtREV

mintax4	mintax4_slow	mintax8	most purposive subset	ribosomal protein	*C. elegans* gene name	corresponding RP	Ortholog ID	Best Model	second best model
X	X	X	X				111829	LG	Blosum62
X	X	X	X	X	rps-27	S27	111830	DCMut*	Dayhoff
X							111841	LG	RtREV
X	X						111844	LG	RtREV
X		X	X				111848	LG	Blosum62
X		X					111849	LG	RtREV
X	X						111857	LG	RtREV
X							111860	LG	Blosum62
X	X	X					111861	LG	JTT
X	X	X					111863	RtREV	LG
X		X	X				111865	LG	CpREV
X	X	X	X				111866	CpREV	LG
X	X	X					111870	LG	RtREV
X	X	X	X	X	rpl-7	L7	111873	LG	RtREV
X	X						111874	LG	RtREV
X	X	X					111889	LG	JTT
X	X						111893	Blosum62	LG
X	X						111894	LG	RtREV
X	X	X	X	X	rps-8	S8	111898	LG	RtREV
X							111900	LG	WAG
X	X						111905	LG	WAG
X	X	X	X	X	rps-24	S24	111917	LG	RtREV
X							111923	LG	JTT
X	X	X	X				111931	LG	RtREV
X	X						111932	LG	JTT
X		X	X	X	rpl-6	L6	111935	LG	WAG
X		X					111938	LG	RtREV
X	X	X	X	X	rpl-20	L18A	111945	LG	RtREV
X	X	X					111948	LG	RtREV
X	X						111950	LG	RtREV
X	X	X					111951	LG	CpREV
X		X	X				111952	LG	Blosum62
X	X						111953	LG	RtREV
X	X	X					111956	LG	JTT
X		X					111958	LG	RtREV
X	X	X					111959	LG	JTT
X	X	X					111960	LG	WAG
X	X	X					111962	Dayhoff	DCMut
X							111963	LG	Blosum62
X							111967	RtREV	LG
X	X						111971	LG	RtREV
X							111972	LG	RtREV

mintax4	mintax4_slow	mintax8	most purposive subset	ribosomal protein	*C. elegans* gene name	corresponding RP	Ortholog ID	Best Model	second best model
X							111978	RtREV	LG
X	X	X					111984	LG	RtREV
X	X			X	rps-2	S2	111988	RtREV	LG
X	X	X	X	X	rpl-7A	L7A	111989	LG	RtREV
X		X	X	X	rpl-24.2	L24 paralog	111992	LG	WAG
X		X					111995	LG	Blosum62
X	X	X	X				111998	LG	JTT
X	X						112002	LG	JTT
X	X	X		X	rpl-3	L3	112003	RtREV	LG
X	X	X					112004	LG	WAG
X							112007	LG	RtREV
X	X	X					112012	LG	RtREV
X	X	X					112013	LG	WAG
X							112021	LG	WAG
X	X						112028	LG	WAG
X	X	X	X				112029	LG	RtREV
X	X	X	X				112034	LG	WAG
X		X	X				112037	LG	WAG
X	X	X	X	X	rpl-2	L8	112038	RtREV	LG
X							112040	CpREV	LG
X	X						112044	LG	WAG
X	X						112047	RtREV	Blosum62
X	X						112056	LG	WAG
X	X	X					112060	JTT	LG
X	X						112064	LG	Dayhoff
X	X	X					112066	LG	JTT
X	X	X	X	X	rps-1	S3A	112067	LG	RtREV
X	X	X					112072	LG	WAG
X							112082	CpREV	JTT
X	X	X					112083	LG	WAG
X	X						112087	LG	WAG
X	X	X					112088	LG	RtREV
X							112096	LG	RtREV
X	X						112097	LG	WAG
X	X	X	X				112102	LG	JTT
X	X						112106	LG	WAG
X	X	X					112107	LG	RtREV
X	X	X					112108	LG	JTT
X	X	X	X	X	rpl-14	L14	112115	LG	RtREV
X	X	X					112123	Dayhoff	DCMut
X	X	X					112124	JTT	LG
X	X	X					112126	RtREV	LG

mintax4	mintax4_slow	mintax8	most purposive subset	ribosomal protein	*C. elegans* gene name	corresponding RP	Ortholog ID	Best Model	second best model
X	X						112129	LG	RtREV
X	X	X					112130	LG	CpREV
X	X	X	X				112137	LG	RtREV
X	X	X					112141	LG	RtREV
X	X	X					112142	LG	WAG
X	X	X	X				112144	LG	JTT
X	X	X					112147	LG	RtREV
X	X	X					112156	LG	JTT
X	X	X					112158	LG	RtREV
X	X	X					112160	LG	JTT
X							112161	RtREV	LG
X	X	X					112163	LG	RtREV
X	X	X					112164	LG	RtREV
X	X	X					112168	LG	WAG
X	X	X					112169	LG	RtREV
X		X					112170	LG	CpREV
X		X	X	X	rpl-29	L29	112178	RtREV	Blosum62
X	X	X	X				112181	LG	JTT
X	X	X	X				112182	LG	RtREV
X							112184	Blosum62	LG
X		X					112187	RtREV	LG
X							112191	LG	WAG
X	X	X	X	X	rpl-1	L10A	112195	LG	RtREV
X	X	X					112199	LG	RtREV
X	X						112201	LG	CpREV
X	X	X	X	X	rps-26	S26	112202	LG	JTT
X	X	X	X				112203	LG	JTT
X	X	X	X	X	rps-6	S6	112204	LG	RtREV
X							112205	Blosum62	VT
X	X	X					112206	LG	WAG
X	X	X					112207	LG	Blosum62
X	X						112211	LG	RtREV
X	X	X					112214	LG	RtREV
X	X						112221	LG	WAG
X	X	X		X	rpl-18	L18	112223	LG	RtREV
X	X	X					112226	LG	WAG
X	X						112231	LG	RtREV
X		X					112233	CpREV	LG
X	X	X					112239	Blosum62	LG
X	X						112240	LG	RtREV
X	X	X	X	X	rpl-9	L9	112242	LG	RtREV
X	X	X					112255	LG	WAG

mintax4	mintax4_slow	mintax8	most purposive subset	ribosomal protein	*C. elegans* gene name	corresponding RP	Ortholog ID	Best Model	second best model
X	X	X					112256	LG	RtREV
X	X	X					112258	LG	RtREV
X	X						112265	LG	RtREV
X							112267	LG	RtREV
X	X	X	X				112271	LG	RtREV
X	X	X					112281	LG	JTT
X	X	X					112284	LG	WAG
X	X						112287	LG	Blosum62
X	X						112288	RtREV	LG
X	X	X					112290	RtREV	LG
X		X	X				112291	WAG	LG
X	X	X					112292	LG	RtREV
X		X					112293	LG	WAG
X							112296	LG	JTT
X	X	X					112297	JTT	LG
X	X	X					112298	LG	WAG
X							112299	LG	WAG
X	X	X					112300	LG	RtREV
X	X	X	X	X	rpl-41	L41	112301	Dayhoff	DCMut
X	X						112302	RtREV	LG
X	X	X	X				112307	LG	RtREV
X							112308	LG	RtREV
X	X	X					112310	LG	RtREV
X	X	X					112311	LG	CpREV
X							112312	LG	WAG
X	X	X					112315	LG	RtREV
X	X						112320	LG	JTT
X	X	X					112321	JTT	LG
X	X	X	X	X	rps-20	S20	112322	LG	RtREV
X	X	X					112324	RtREV	LG
X							112325	LG	RtREV
X							112330	LG	JTT
X	X						112337	LG	RtREV
X	X	X					112339	LG	WAG
X	X						112342	LG	RtREV
X	X	X					112345	WAG	VT
X	X						112349	LG	RtREV
X	X	X					112350	LG	JTT
X	X	X					112354	LG	RtREV
X	X	X	X	X	rps-5	S5	112355	LG	RtREV
X	X	X	X	X	rpl-30	L30	112358	LG	RtREV
X	X	X	X	X	rpl-15	L15	112359	LG	RtREV

mintax4	mintax4_slow	mintax8	most purposive subset	ribosomal protein	*C. elegans* gene name	corresponding RP	Ortholog ID	Best Model	second best model
X	X	X	X				112366	WAG	LG
X	X	X					112367	LG	WAG
X	X	X					112371	RtREV	LG
X	X	X					112372	LG	RtREV
X	X						112374	LG	RtREV
X	X						112375	LG	WAG
X	X	X	X				112376	LG	Dayhoff
X	X	X	X				112379	LG	RtREV
X		X					112380	LG	RtREV
X							112381	LG	Blosum62
X	X	X					112382	WAG	LG
X	X	X	X				112386	LG	RtREV
X	X						112388	LG	RtREV
X	X	X	X	X	rpl-36	L36	112394	RtREV	LG
X	X	X					112399	LG	JTT
X		X					112402	LG	RtREV
X		X					112403	LG	RtREV
X	X	X	X	X	rps-12	S12	112414	LG	WAG
X	X	X					112415	LG	RtREV
X	X	X	X	X	rpl-26	L26	112417	LG	RtREV
X	X						112421	LG	WAG
X	X	X					112422	LG	JTT
X	X						112423	LG	RtREV
X	X	X	X	X	rpl-27	L27	112425	LG	RtREV
X	X	X					112427	LG	JTT
X							112430	LG	RtREV
X		X					112432	LG	WAG
X	X	X	X				112433	LG	JTT
X	X	X	X	X	rpl-16	L13A	112436	LG	RtREV
X	X	X					112438	LG	RtREV
X	X	X					112443	LG	JTT
X	X	X	X				112448	LG	RtREV
X	X	X	X				112449	LG	RtREV
X	X						112450	LG	RtREV
X	X	X	X	X	rps-21	S21	112451	LG	Blosum62

sum 410 307 272 101 51

* = special case: always assigned to partition "Dayhoff"
