# Supplementary material for: Transcriptome Data Reveal Syndermatan Relationships and Suggest the Evolution of Endoparasitism in Acanthocephala via an Epizoic Stage
Source: PLoS One. 2014 Feb 10;9(2):e88618. doi: 10.1371/journal.pone.0088618 (PMC3919803; doi:10.1371/journal.pone.0088618)
Supplement: Table S4 — Support values for internal nodes, position of Turbanella and position of Paraplanocera spec. and M. lignano obtained by all analyses. Statistical support values as obtained from four different phylogenetic reconstruction programs are given for the phylogenomic datasets, defined in detail in Material and Methods. Taxon abbreviations are: S = Seison, A = Acanthocephala, B = Bdelloidea, M = Monogononta, Syn = Syndermata, G = Gnathostomulida, T = Turbanella ambronensis. (*) Possible phylogenetic positions for the gastrotrich Turbanella ambronensis ( = "Turbanella ") are: 1 = sister to (Platyhelminthes + Syndermata + Gnathostomulida), 2 = sister to Gnathifera, 3 = trichotomy (Mollusca,Turbanella,(Platyhelminthes+Gnathifera)), 4 = sister to Platyhelminthes, 5 = clade (Turbanella + Gnathostomula) sister to Syndermata, 6 = sister to Syndermata, 7 = clade (Turbanella + Gnathostomula) sister to Platyhelminthes. (PDF) [file pone.0088618.s005.pdf]

**Table S4 - Support values for internal nodes, position of *Turbanella* and position of *Paraplanocera* spec. and *M. lignano* obtained by all analyses**

S = *Seison* , A = Acanthocephala, B = Bdelloidea, M = Monogononta, Syn = Syndermata, G = Gnathostomulida, T = *Turbanella ambronensis*

*: possible positions for the gastrotrich *Turbanella ambronensis* (="*Turbanella*"): 1 = sister to (Platyhelminthes + Syndermata + Gnathostomulida), 2 = sister to Gnathifera, 3 = trichotomy (Mollusca,*Turbanella* ,(Platyhelminthes+Gnathifera)), 4 = sister to Platyhelminthes, 5 = clade (*Turbanella* + *Gnathostomula* ) sister to Syndermata, 6 = sister to Syndermata, 7 = clade (*Turbanella* + *Gnathostomula* ) sister to Platyhelminthes

| | | | Lemniscea | Pararotatoria | Hemirotifera | Syndermata | Gnathifera | | | |
|---|---|---|---|---|---|---|---|---|---|---|
| **Pyhlogenomic dataset** | **program** | **number of partitions** | **A+B** | **S+A** | **(S+A+B)** | **(S+A+B)+M** | **Syn+G** | **Syn+(G+T)** | **Position of *Turbanella* *** | **Topology of *Paraplanocera* spec. + *M. lignano*** |
| mintax4 | MrBayes | 7 | / | 1,0 | 1,0 | 1,0 | 1,0 | / | 1 | Para basal |
| mintax4 | PhyloBayes | 1 | / | 1,0 | 1,0 | 1,0 | 0,84 | / | 2 | Macro basal |
| mintax4 | RAxML | 8 | / | 100 | 87 | 100 | 76 | / | 1 | Para sister to Macro |
| mintax4 | Treefinder | 8 | / | 100 | 82 | 100 | 87 | / | 3 | Para sister to Macro |
| mintax4_noRPs | MrBayes | 7 | / | 1,0 | 1,0 | 1,0 | 1,0 | / | 4 | Macro basal |
| mintax4_noRPs | PhyloBayes | 1 | / | 1,0 | 1,0 | 1,0 | 0,89 | / | 4 | Macro basal |
| mintax4_noRPs | RAxML | 8 | / | 100 | 97 | 100 | 71 | / | 4 | Macro basal |
| mintax4_noRPs | Treefinder | 8 | / | 99 | 96 | 100 | 90 | / | 4 | Macro basal |
| mintax8 | MrBayes | 7 | / | 1,0 | 1,0 | 1,0 | 1,0 | / | 1 | Para basal |
| mintax8 | PhyloBayes | 1 | / | 1,0 | 1,0 | 1,0 | 0,86 | / | 2 | Macro basal |
| mintax8 | RAxML | 8 | / | 100 | 96 | 100 | 54 | / | 1 | Para sister to Macro |
| mintax8 | Treefinder | 8 | 0 | / | 99 | 100 | 56 | / | 2 | Para sister to Macro |
| mintax8_noRPs | MrBayes | 7 | / | 1,0 | 1,0 | 1,0 | 1,0 | / | 4 | Macro basal |
| mintax8_noRPs | PhyloBayes | 1 | / | 1,0 | 1,0 | 1,0 | 0,96 | / | 4 | Macro basal |
| mintax8_noRPs | RAxML | 8 | / | 100 | 97 | 100 | 80 | / | 4 | Macro basal |
| mintax8_noRPs | Treefinder | 8 | / | 100 | 99 | 100 | 91 | / | 4 | Macro basal |
| most purposive subset | MrBayes | 1 | / | 1,0 | 0,99 | 1,0 | / | 1,0 | 5 | Para basal |
| most purposive subset | RAxML | 1 | / | 100 | 49 | 100 | / | 56 | 5 | Para basal |
| most purposive subset | Treefinder | 1 | / | 100 | 58 | 100 | / | 73 | 5 | Para basal |
| most purposive subset_noRPs | MrBayes | 1 | / | 1,0 | 1,0 | 1,0 | / | / | 1 | Para basal |
| most purposive subset_noRPs | RAxML | 1 | / | 69 | 47 | 95 | / | 25 | 5 | Para basal |
| most purposive subset_noRPs | Treefinder | 1 | / | 91 | 71 | 94 | / | / | 6 | Para basal |
| mintax4_slow | RAxML | 8 | / | 99 | 85 | 100 | 79 | / | 1 | Para basal |
| mintax4_4Synd | RAxML | 1 | / | 68 | 64 | 99 | 65 | / | 1 | Para basal |
| mintax4_4Synd-DS | RAxML | 1 | / | 86 | 86 | 95 | / | / | 7 | Para basal |
